# Can PSMA PET/CT help in dose-tailoring in post-prostatectomy radiotherapy?

**DOI:** 10.3389/fonc.2023.1268309

**Published:** 2023-09-20

**Authors:** Ambroise Champion, Daniel Rudolf Zwhalen, Christoph Oehler, Daniel Taussky, Stephanie G. C. Kroeze, Irene A. Burger, David Benzaquen

**Affiliations:** ^1^ Radiation Oncology, Hôpital de La Tour, Meyrin, Switzerland; ^2^ Department of Radiation Oncology, Kantonsspital Winterthur, Winterthur, Switzerland; ^3^ Department of Radiation Oncology, Centre Hospitalier de l’Université de Montréal, Montréal, QC, Canada; ^4^ Department of Radiation Oncology Kantonsspital Aarau and Baden, Kantonsspital Aarau, Aarau, Switzerland; ^5^ Department of Nuclear Medicine, Kantonsspital Baden, Baden, Switzerland; ^6^ Department of Nuclear Medicine, University Hospital Zürich, University of Zürich, Zurich, Switzerland

**Keywords:** prostate cancer, postoperative radiotherapy, PSMA-PET, PSA, dose ecalation

## Abstract

There are few randomized trials to evaluate the use of PSMA-PET in the planning of post-prostatectomy radiotherapy. There are two unresolved questions 1) should we increase the dose to lesions visible on PSMA-PET 2) can we reduce dose in the case of a negative PSMA-PET. In this review, we summarize and discuss the available evidence in the literature. We found that in general, there seems to be an advantage for dose-increase, but ta large recent study from the pre-PSMA era didn’t show an advantage for dose escalation. Retrospective studies have shown that conventional doses to PSMA-PET-positive lesions seem sufficient. On the other hand, in the case of a negative PSMA-PET, there is no evidence that dose-reduction is possible. In the future, the combination of PSMA-PET with genomic classifiers could help in better identify patients who might benefit from either dose- de-or -increase. We further need to identify intraindividual references to help identify lesions with higher aggressiveness.

## Introduction

Prostate-specific membrane antigen (PSMA) positron emission tomography (PET) has led to a shift in the management of many different clinical situations in prostate cancer (1). It is foreseeable that as we gain more experience with PSMA-PET, with its better accuracy to detect recurrence, we will be able to improve the management of biochemical recurrence after prostatectomy (2, 3). The dilemma in the era of evidence-based medicine is that there are very few randomized trials to evaluate the use of PSMA in the planning of radiotherapy treatment (4). There are essentially two unresolved questions that we try to address in this review: 1) can lesions visible on PSMA-PET benefit from dose-escalation (5, 6). 2) can we reduce dose in the case of a negative PSMA-PET (7). In this review, we summarize and discuss the available evidence in the literature on whether PSMA-PET can help in postoperative radiotherapy by dose-tailoring, either by focal de-escalation or escalation of the applied radiation dose.

## The value of PSMA-PET in staging for biochemical recurrence after radical prostatectomy

In primary, untreated prostate cancer, the randomized proPSMA trial has shown prospectively, that [^68^Ga]-PSMA-11 PET-CT is more accurate in staging prostate cancer than conventional imaging (Computed tomography (CT) and bone scan) (8). There is so far no randomized trial to show such an advantage in the postoperative setting, except for a large number of retrospective studies conducted on large numbers of patients. Thus, in a scoping review for patients with biochemical recurrence, Valle et al. (9), summarized 45 articles with over 240 patients imaged with [^18^F]-Fluciclovine and over 3000 patients imaged with [^68^Ga]-PSMA-11 and showed that PSMA-PET imaging resulted in a higher detection rate of local recurrences or metastases than conventional imaging or other PET tracers at low PSA values. Depending on the tracer used in PSMA-PET, changes in treatment management occurred in 40–77% of patients (9). Whether or not one finds such lesions on PSMA-PET depends heavily on the PSA at the time of imaging. In a large multicenter study of more than 2000 patients published in 2022 (10), the detection rate was 44% (44/105) when PSA levels were < 0.25 ng/mL. In conclusion, PSMA-PET has been shown to improve the detection rate of recurrence after prostatectomy compared to conventional imageing but how to implement the findings into daily practice has not been established yet.

## Do we need to increase the dose in postoperative radiotherapy to PSMA-PET-positive lesions?

### The pre PSMA-PET era

Retrospective studies found an advantage for dose-escalation. Two early retrospective studies as well as later studies, including a more recent one, found that higher doses of salvage RT might reduce progression rates (11, 12). They showed that an EQD2 of 68 Gy or 70.2 Gy in 1.8 Gy fractions resulted in the most favorable results, but only in the subgroup with positive surgical margins (n=98). King, in his extensive review (13), found that in patients treated with radiotherapy only without androgen deprivation therapy (ADT), that the radiotherapy dose and PSA level prior to RT were the only significant factors for recurrence (PSA ≥0.2 ng/mL after salvage radiotherapy). He reported a very well fit sigmoidal relationship between dose and recurrence with an improvement in recurrence-free survival by 2.0% per additional Gy given [95%CI: 1.1–3.2]. He calculated that a dose of 70 Gy would achieve a 58.4% rate of RFS vs. 38.5% for 60 Gy. However, he could not identify a subgroup of patients who might benefit from dose-escalation. Similar findings were described even ten years earlier by Ohri et al. (14) in their systematic review and regression meta-analysis with radiobiological modelling. They stated that the maximum achievable 5-year recurrence-free survival following salvage RT appears to be between 70% and 80%. This suggests that a portion of patients who receive salvage RT already have occult extra-pelvic disease. Indeed, as reported by King (13), Ohri et al. (14) found that the 5-year recurrence-free survival increased by 2.5% per Gy (95% CI: 1.0–4.0%) and that recurrence-free survival decreased with increasing pre-radiotherapy PSA, (–18.1% per 1 ng/mL increase, 95% CI: [–29.2% to –7.0%]). A smaller (n=144) prospective study had shown an advantage for a dose increase from 66 to 72 Gy in the salvage setting only in in patients with a higher Gleason score (8–10). However, a longer follow-up period of 4-years revealed no advantage of dose escalation for biochemical PFS (15). In this line a recently published, much larger, prospective randomized study [SAKK 09/10 (n=350)] (16) showed no advantage for dose-escalation to 70 Gy compared to 64 Gy. But higher dose resulted in higher gastrointestinal toxicity. Surgical margin status was not analyzed as a prognostic factor in this study. In this trial, patients were treated to the prostate bed only, no routine PSMA-PET was performed and no androgen deprivation therapy (ADT) was given (16).

In conclusion, although a large randomized trial found no advantage for dose escalation to 70Gy, several retrospective studies showed an advantage.

### PSMA-PET era

The above-mentioned data seem in in contradiction to the results with PSMA-PET: conventional doses to PSMA-PET-positive lesions seem sufficient. Only Rowe et al. (17) found a high rate of persistent local disease on PSMA-PET within the radiotherapy field after salvage radiotherapy. They reported that 7/32 patients (22%) had such a recurrence and that six of these local recurrences were within 100% of the prescribed isodose of 66.6-72 Gy (17). The local recurrence rate in this study was much higher than the one reported by Byrne et al. (18) who found a PSMA-PET determined local failure rate within the radiation-field in only 2/50 (4%) of patients treated with prostate bed radiotherapy, despite even slightly lower doses of 64-68 Gy. Another study by Solomonidou et al. (19) using PSMA-PET before salvage radiotherapy also found a lower in-field recurrence rate and showed that recurrences appeared in only 1/16 patients inside of the RT-field treated with 66-70 Gy. This in patients who had a PSMA-PET before salvage radiotherapy and a second one at PSA relapse. The above findings show that in the PSMA-era in-field recurrences after salvage radiotherapy are rare. Interestingly, the study by Byrne et al. (18) found that salvage radiotherapy with the help of PSMA-CT to the prostate bed (n=310) or bed with lymph nodes (n=99) lead to an excellent in-field control (4% vs. 6%) and a similar control rate for PSA relapse of 71% and 70%, respectively, despite a higher rate of ADT in the group with nodal RT (13% vs 92%). Isolated nodal failure occurred in 1/3 of the patients with prostate bed therapy only but was rare in the group with prostate bed and pelvic node irradiation. Nevertheless, although local recurrence after radiotherapy is rare, there is some promising data on dose-escalation to >72 Gy on PSMA-positive lesions. Vogel et al. (20) used a simultaneous-integrated boost for PSMA-PET-positive lesions to 76.5 Gy in 2.25 Gy vs. 68 Gy in 2 Gy fractions. They found a better PSA-response rate for patients treated with dose-escalation. Only 1/7 patients who had a PSMA-PET for recurrence after radiotherapy had a local recurrence. It wasn’t clear if this recurrence was in the boost-volume or not. Another study investigated dose-escalation in patients with a positive MRI after prostatectomy. Benziane-Ouaritini (21) et al. found that dose of ≥72 Gy showed better progression free survival on multivariate analysis. Others are studying dose-escalation to the lymphnodes (Prospser 1) (22), in patients with insufficient PSA response after 50 Gy. It therefore seems that the benefit of dose-escalation to positive lesions on PSMA-PET is not clear.

## Are patients with a negative PSMA-PET ready for dose-tailoring?

There are two possible explanations in the case of an elevated PSA post-prostatectomy but absence of radiologic disease on PSMA-PET. Either the patient is metastatic and needs to ADT to radiotherapy. Or, there is non-visible local disease and dose-reduction would risk undertreating the patient. According to the recently published Advanced Prostate Cancer Consensus Conference (APCCC) 2022 (23), the decision on how to best treat these patients depends on several factors. But radiation- dose-reduction was not an option.

While the experts in APCC Conference didn’t discuss to not treat patients with a negative PSMA-PET, others, especially in Australia, argue that there is no clear opinion on how to treat patients with negative PSMA-PET (24).

Another possibility would be to spare the prostate bed and treat the lymphnodes only. This because post-radiotherapy recurrences in PSMA-PET negative patients occurred in 44% in the lymphnodes (25). There are a few studies with a smaller number of patients, which focused on the prognostic importance of a negative PSMA-PET, summarized in **Table 1**. For example, Scharl et al. (25) in their retrospective multicenter review compared 173 patients with a negative PSMA-PET with 168 patients with only local disease in the prostatic bed. Univariate analysis for biochemical progression-free survival (PFS) post radiotherapy showed that PSMA-PET negative patients had better PFS than PET/CT positive patients (80.8% vs. 71.6% p = 0.019). However, risk factors were not even distributed in both groups, PSA doubling time was significantly shorter in the PSMA-PET positive group. The authors concluded, that salvage RT should not be withhold from patients with negative PSMA PET/CT. Similarly, Emmet et al. (27) found that patients with a negative PSMA-PET had the best 3-yr freedom from progression (FFP; 82.5%) compared to patients with localized disease. even though the patients with a negative PSMA-PET received smaller radiotherapy fields and were less likely to receive ADT. This is in line with an earlier publication of Emmet et al. (26) reporting that in PSMA-PET negative patients treatment response was not different compared to patients with a positive PSMA-PET in the prostate-bed. This in patients with a PSA 0.05-1.0 ng/mL, treatment response defined as both PSA ≤ 0.1 ng/mL and >50% reduction in PSA. Another study (29) showed a nearly identical outcome in PSMA-PET positive and negative patients. In a retrospective study from 2 German centers, Schmidt-Hegemann et al. (29) showed that PSMA-negative and -positive patients had the same outcome when treated with salvage radiotherapy alone without ADT. In this study, 53% (48/90) of the patients had a negative PSMA. The median follow-up in this study was short (23 months) and therefore results on outcome should be considered cautiously. Biochemical recurrence in patients treated without ADT was not different whether the PSMA was negative or positive (29). Interestingly, in contradiction to the other studies, in a recent study, Solomonidou et al. (19) found that patients with a PSA of <0.2 ng/mL and with localized disease on PSMA-PET had worse biochemical recurrence-free survival than patients with a negative PSMA-PET. Some argue that a negative PSMA-PET doesn’t require immediate treatment and that progression to treatment in initially un-treated patients with a negative PSMA-PET is only 15-60% (27, 30). In conclusion, it seems that a negative PSMA-PET isn’t necessarily a sign for better outcome after salvage radiotherapy. It seems that results are similar in patients treated with a negative and positive PSMA-PET. Therefore, so far there is no scientific evidence for dose-de-escalation or omission of treating the prostate-bed in this context.

**Table 1:** Publications with a negative PSMA-PET.

Author	N= with neg. PSMA	Outcome measure	outcome	Follow-up in months	Tracer	Journal/year
Emmet	27	treatment response: both PSA ≤ 0.10 ng/mL *and* greater than 50% reduction from pretreatment level	86% treatment response	Median 10.5 (IQR 6–14)	^68^Ga-PSMA	J Nucl Med 2017 (26)
Emmet	90	FFP: PSA remaining ≤0.2 ng/mL above nadir	negative PSMA FFP 82.5% vs PSMA pos. in prostate bed 79%	38 (IQR 31–43)	^68^Ga-PSMA	J Nucl Med 2020 (27)
Scharl	173	nadir after SRT + 0.2 ng/ml) Local control: absence of local recurrence	BPFS lower in the PET-negative group (71.6%) than in the locally PET-positive group (80.8%, p = 0.019) Cox regression: no difference	median 28.0 (IQR 25.7–30.3)	^68^Ga-PSMA-11 ^18^F-PSMA-1007 ^68^Ga-PSMA-I&T ^18^F-PSMA-DCFPyL ^18^F-PSMA-rhPSMA-	Radiother Oncol. 2023 (25)
Schmidt-Hegemann	48	BRFS (PSA ≤ 0.2 ng/mL)	No difference between patients with and without PET-positive findings (78% vs. 82%, P=0.39). addition of ADT without effect (P = 0.41)	Median 23 (range 1–47)	^68^Ga-PSMA	J Nucl Med 2019 (28)
Solomonidou	192	BRFS: PSA nadir after sRT + 0.2 ng/ ml or death of any cause	Local failure in PSMA-PET/CT (yes vs. no) HR 0.459 (0.22–0.94 p=0.04)	median 31.1 (IQR 20–44)	^68^Ga-PSMA-11 ^68^Ga-PSMA-I&T ^18^F-PSMA-1007 ^18^F-siPSMA-14 ^18^F-SMArhPSMA	Eur J Nucl Med Mol Imaging 2023 (19)

## Are there other factors than PSMA-PET that could help identify a specific group for dose-tailoring?

In a smaller open-label randomised controlled trial, the use of ^18^F-fluciclovine-PET/CT for the decision-making process in radiotherapy resulted in a 12% improvement in event-free survival at 3 years. An event was defined as a PSA of 0.2 ng/mL higher than the nadir after radiotherapy, followed by another rise or persistent PSA, or failure on imaging or digital rectal examination (31).

If one may not rely only on PSMA-PET for dose tailoring, maybe one could use PSMA-PET in conjunction with clinical factors. Patients with a low PSA and positive surgical margins are probably the ones who benefit most from salvage-radiotherapy. In these patients with low PSA and positive surgical margins, the benefit from dose – escalation might be biggest because of the higher chance of local recurrence.

The importance of treating patients in the postoperative setting at a low PSA level has been recently shown in a multicenter retrospective study of >2500 patients (32). It was found that all-cause mortality was higher for patients with a prior radiotherapy PSA of >0.25 ng/mL compared to ≤0.25 ng/mL. Other studies in the era of PSMA-PET have shown similar results (33–35).

A multi-institutional database for patients treated with salvage RT as well as in the adjuvant setting has shown that positive margins have the best positive predictive factor for biochemical control after postoperative radiotherapy (36). Similar results were reported in a much earlier EORTC 22911 study by Van der Kwast et al. (37), who showed that positive surgical margins were the only factor predicting for the benefit of adjuvant radiotherapy. Patients who had positive surgical margins and were treated with adjuvant radiotherapy had a 5-year recurrence free survival of 78% vs. 49% for patients in the control arm receiving radiotherapy in a non-adjuvant setting No benefit was found for the other two inclusion criteria; extracapsular extension and seminal vesicle invasion. Adjuvant radiotherapy had no effect on outcome in patients with a high Gleason score or seminal vesicle involvement if the surgical margins were negative. These patients seemed to have already metastasized. It must be noted that a large (>11,500 patients) retrospective series showed the effect of salvage radiotherapy in more aggressive margin-negative patients (38). But this effect might have been influenced by the use of ADT or other risk factors.

## PSMA as a biomarker?

If we have no clear data on tailoring radiation-therapy dose according to PSMA-PET findings, maybe PSMA can help as a biomarker. Spohn et al. (39), in a multicenter study consisting of 235 patients with recurrence on PSMA-PET after prostatectomy, found that PSMA positive recurrence with an SUVmax (standardized uptake value) in the 4^th^ quartile was a marker for biochemical recurrence after salvage radiotherapy (HR 2.3, p=0.022). They further found that biochemical recurrence-free survival at 2 years in patients with recurrence confined to the prostate bed was 80%. This led to their conclusion that patients with prostate bed -confined disease might benefit from intensification of local treatment instead of systemic treatment. Other studies have found a correlation between quantitative PSMA uptake and cancer aggressiveness (40, 41). But it is presently not possible to use PSMA as a reproducible biomarker. Values between different centers can’t be compared. Differences in SUV between centers are too big due to differences in tracers, scanners, protocols, and imaging time points. Although validation of genomic classifiers is still needed (42), a combination with PSMA-PET could further help identify more favorable subgroups. Patients in SAKK 09/10 with a Decipher score of low to intermediate, had a 5-year freedom from biochemical progression (FFBP) of 71% (43). This is similar or a bit inferior to the ~ 80%, in the most favorable prognostic factor groups in the PSMA-PET studies (39).

Another possibility may be the use of PSMA as a biomarker on circulating tumor cells (CTCs). So far, in small studies, a high fraction of PSMA-negative CTCs has been shown to be a negative prognostic factor in metastatic hormone-resistant cancers (44, 45).

In the future, intraindividual references (e.g., PSMA Score with respect to uptake in liver/blood pool for ^68^Ga-PSMA-11) could help to identify lesions with higher aggressiveness that could benefit from dose-escalation. In the mean-time, PSMA-PET findings have already been incorporated into nomograms (35).

In conclusion, while PSMA can serve as a road map for dose-tailoring in radiotherapy in the postoperative salvage setting, we couldn’t find a scientific rationale for dose reduction in PSMA-PET negative or dose increase in PSMA-PET positive patients. Because some studies show cancer persistence in the radiotherapy field after PSMA-PET guided radiotherapy, several ongoing studies are pursuing the avenue of dose-escalation with PSMA—imaging, especially with hypofractionation (46, 47). Patients with positive surgical margins and low-PSAs have the highest risk of local recurrence only and might benefit most from dose-escalation.

## Data availability statement

The original contributions presented in the study are included in the article/supplementary material, further inquiries can be directed to the corresponding author/s.

## Author contributions

AC: Writing – original draft, Writing – review & editing. DZ: Writing – original draft, Writing – review & editing. CO: Writing – original draft, Writing – review & editing. DT: Writing – original draft, Writing – review & editing, Conceptualization. SK: Writing – original draft, Writing – review & editing. IB: Writing – original draft, Writing – review & editing. DB: Writing – original draft, Writing – review & editing.
